# Association of Procalcitonin, C-reactive Protein, and White Blood Cell Count With Acute Exacerbations of Chronic Obstructive Pulmonary Disease: A Cross-Sectional Study

**DOI:** 10.7759/cureus.101474

**Published:** 2026-01-13

**Authors:** Heena Shishodia, Rati Mathur

**Affiliations:** 1 Biochemistry, Sawai Man Singh (SMS) Medical College and Attached Hospitals, Jaipur, Jaipur, IND

**Keywords:** c-reactive protein, dyspnea, inflammation, procalcitonin, spirometry

## Abstract

Background

Acute exacerbations of chronic obstructive pulmonary disease (AECOPD) are characterized by heightened airway and systemic inflammation. Inflammatory biomarkers such as white blood cell (WBC) count, procalcitonin (PCT), and C-reactive protein (CRP) may serve as useful indicators of the presence of acute exacerbations and assist in clinical assessment and management. The present study aimed to evaluate variations in these inflammatory markers in relation to the clinical status of patients during AECOPD.

Methods

A cross-sectional study was conducted in the Department of Biochemistry and Respiratory Medicine at Sawai Man Singh Hospital, Jaipur, India, and included 80 spirometry-confirmed COPD patients diagnosed according to the Global Initiative for Chronic Obstructive Lung Disease (GOLD) 2023 guidelines. Categories B and E patients, as per GOLD 2023 guidelines, were included for a direct comparison of acute exacerbation. Demographic characteristics, comorbidities, GOLD classification, Modified Medical Research Council dyspnea grade (mMRC), and smoking history were recorded. Levels of PCT, CRP, and WBC count were compared across Categories B and E and dyspnea grades.

Results

The cohort of 80 patients had a mean age of 65.7 years with a standard deviation of 12.2 years. There were 66 (82.5%) males and 14 (17.5%) females. Hypertension and diabetes were the most common comorbidities. Based on GOLD criteria, 50 (62.5%) were classified as category E and 30 (37.5%) as category B. Category E patients had significantly higher levels of PCT, CRP, and WBC compared to category B. Most patients belonged to mMRC grades 2 and 3; however, inflammatory marker levels did not differ significantly across mMRC grades.

Conclusion

PCT, CRP, and WBC levels were significantly elevated in patients with more severe COPD, particularly those in GOLD category E, indicating their usefulness in assessing disease severity and inflammatory burden. Incorporating these biomarkers may enhance the evaluation of COPD exacerbation severity and guide management strategies in COPD.

## Introduction

Chronic obstructive pulmonary disease (COPD) is a progressive respiratory disorder characterized by persistent airway inflammation and airflow obstruction. Globally, COPD affects approximately 10.6% of the population, accounting for nearly 480 million cases worldwide [1]. In India, data from the Longitudinal Aging Study of India (LASI) report a prevalence of 2.1% among individuals aged 45 years and older, highlighting a substantial national disease burden [2]. The underlying pathophysiology involves a complex immune response, predominantly mediated by Th1-type inflammation triggered by exposure to toxins, microbes, or autoimmune mechanisms, with disease chronicity sustained by Th2-type immune responses [3].

Clinically, patients commonly present with chronic cough with or without sputum production, dyspnea, and chest tightness, symptoms that often result in marked limitation of daily physical activities and reduced quality of life [3]. In addition to its chronic progressive course, COPD is frequently complicated by acute exacerbations of COPD (AECOPD), which are episodes of acute worsening of respiratory symptoms. Nearly 30-50% of COPD patients experience one or more exacerbations, events that substantially increase healthcare utilization and are associated with higher morbidity and mortality [4].

Disease severity and clinical stratification of COPD are guided by the Global Initiative for Chronic Obstructive Lung Disease (GOLD) 2023 guidelines, which emphasize symptom burden and exacerbation history for patient classification [5]. However, given the central role of inflammation in both stable disease and acute exacerbations, there has been growing interest in the use of systemic inflammatory biomarkers to better characterize disease activity [5-7].

Among the commonly studied biomarkers are procalcitonin (PCT), C-reactive protein (CRP), and white blood cell (WBC) count, because they reflect systemic inflammation in acute exacerbations [6]. Despite their potential clinical utility, limited data are available, particularly from the Indian population, regarding the relationship between serum PCT, CRP, and WBC levels and acute exacerbations in COPD [7-11]. Therefore, the present study was undertaken to assess variations in serum PCT, CRP, and WBC in relation to the acute exacerbations in COPD. The objectives were to evaluate baseline levels of these inflammatory biomarkers and to determine their association with AECOPD, with the aim of improving risk stratification and clinical management.

## Materials and methods

A cross-sectional study was done in the Department of Biochemistry and Respiratory Medicine in Sawai Man Singh Hospital, Jaipur, India. After taking approval from the Institutional Ethical Committee (1090 MC/EC/2023, dated 22/12/2023), the study was conducted over a period of one year, from September 2023 until August 2024, on spirometry-confirmed COPD cases following GOLD 2023 guidelines [5] who attended the outpatient department of our hospital and were referred to the respective departments for investigations and management.

Inclusion criteria

The study enrolled adult patients (>=18 years) with confirmed COPD, as defined by established diagnostic criteria [5], who presented to the outpatient department of our hospital and were either managed on a day-care basis or admitted to the Department of Respiratory Medicine. Patients classified under GOLD 2023 category B (severe disease), who were managed on an outpatient basis, and category E, who presented with acute exacerbations requiring hospital admission, were included in the study. Enrollment was restricted to individuals who voluntarily provided written informed consent. Category B patients were stable at the time of enrolment in the study and did not have an acute exacerbation attack; rather, they had a history of an episode of AECOPD. Category E patients were in the acute exacerbation attack and AECOPD was defined as an acute worsening of respiratory symptoms beyond normal day-to-day variation, characterized by increased dyspnea, cough, and/or sputum volume or purulence, necessitating a change in regular medication (such as initiation or escalation of bronchodilators, corticosteroids, and/or antibiotics), in accordance with GOLD 2023 criteria. Spirometry was not performed during the acute exacerbation phase. Patients were enrolled during acute exacerbations based on clinical criteria, while spirometric assessment was deferred until clinical stabilization, in accordance with standard guidelines.

Exclusion criteria

Patients aged <18 years were excluded from the study. Individuals with active lung malignancy or those receiving long-term antibiotic therapy were not eligible. Patients on long-term oxygen therapy (LTOT), those in the immediate post-operative or post-surgical period (surgery within the last month), and individuals with immunocompromised states (including HIV infection, chronic steroid use, chemotherapy, or organ transplantation) were excluded. Patients who declined to provide written informed consent were not included in the study.

Sample size

The sample size was determined using reference data from Gao et al. [12], who reported mean serum PCT levels of 2.07 ± 5.57 ng/mL and hs-CRP levels of 3.66 ± 3.95 mg/L in patients with AECOPD. Based on these values, and targeting an accuracy of ±1.25 ng/mL for PCT or ±1.25 mg/L for hs-CRP at a 5% significance level, the minimum required sample size was calculated to be 77. To improve the robustness of the study, the final sample included 80 participants.

The study was initiated after informing the patients and taking written informed consent from the eligible patients. Patients’ demographic and clinical details were noted in a study form. Severity of COPD was assessed by GOLD 2023 staging [5], while dyspnea severity was assessed by Modified Medical Research Council (mMRC) scale, which categorizes the patients into grades 1-5, with grade 5 having higher severity [13].

All routine investigations performed as part of standard hospital care were recorded for analysis, including complete blood count (CBC), random and fasting blood sugar (mg/dL), renal function tests comprising blood urea and serum creatinine (mg/dL), and liver function tests (serum bilirubin, aspartate aminotransferase (AST), alanine aminotransferase (ALT), and alkaline phosphatase (ALP) in IU/L). Sputum investigations were carried out in patients producing sputum and included Gram staining and culture-sensitivity testing wherever clinically indicated.

A chest X-ray (posteroanterior view) was performed in all hospitalized patients to evaluate radiological changes and exclude alternative diagnoses such as pneumonia or pneumothorax. Erythrocyte sedimentation rate (ESR) was not routinely performed and was therefore not included in the analysis. Inflammatory biomarkers assessed included total leukocyte count (TLC) (cells ×10^9^/µL), PCT (ng/mL), and CRP (mg/L). PCT was measured using a chemiluminescent immunometric assay on the Vitros 5600 Integrated System (Ortho Clinical Diagnostics, Raritan, NJ, USA) with a normal range of <0.1 ng/mL. CRP was measured using a fixed-point immunorate immunoassay on the same platform and reported with values <50 mg/L considered normal and ≥50 mg/L indicative of systemic inflammation.

Statistical analysis

Data were presented in tables and figures as N(%) or mean ± SD. Data normality was checked using the Shapiro-Wilk test. An independent t-test was used to compare quantitative variables. IBM SPSS Statistics for Windows, Version 25 (Released 2017; IBM Corp., Armonk, New York, United States) was used. A p-value <0.05 was considered statistically significant.

## Results

A total of 80 COPD cases were enrolled. The study population had an average age of 65.7 ± 12.2 years, indicating that most participants were elderly. The cohort was predominantly male (66, 82.5%), with females representing 14 (17.5%) cases. Among the comorbid conditions, hypertension and type II diabetes mellitus were the most common. The median duration of COPD among study patients was six years, and the median number of exacerbations was three (Table 1).

**Table 1:** Demographic and clinical characteristics of study patients Values are written in mean ± SD or n (%) or median (25th-75th quartile). COPD: chronic obstructive pulmonary disease; IQR: interquartile range

Parameters	Values
Age, mean ± SD, years	65.7 ± 12.2
Males	66 (82.5%)
Females	14 (17.5%)
Comorbidities	
Type II diabetes mellitus	12 (15%)
Coronary artery disease	1 (1.25%)
Cerebrovascular accident	2 (2.5%)
Hypertension	18 (22.5%)
Cor pulmonale	5 (6.25%)
Duration of COPD, median (IQR), years	6 (3-8)
Number of exacerbations, median (IQR)	3 (2-5)

The smoking status distribution showed that 40 patients (50.00%) were former smokers, 29 patients (36.25%) were current smokers, and 11 patients (13.75%) were non-smokers.

Mean values of respiratory rate, pulse rate, systolic blood pressure, diastolic blood pressure, and SpO_2_ were 20.54 ± 4.3 per minute, 96.69 ± 15.17 per minute, 126.95 ± 15.88 mm Hg, 78.58 ± 10.51 mmHg, and 96.5 ± 1.3%. The clinical classification of patients based on the GOLD disease grading system revealed that 50 cases (62.50%) fell into Group E, while the remaining 30 cases (37.50%) were categorized under Group B (Table 2).

**Table 2:** GOLD 2023 disease severity grading of COPD Only categories B and E patients were included in the study. GOLD: Global Initiative for Chronic Obstructive Lung Disease; COPD: chronic obstructive pulmonary disease; mMRC: Modified Medical Research Council; CAT: COPD Assessment Test

GOLD Group	Symptom Burden	Assessment Tool	Exacerbation History (Previous 12 Months)	N (%)
Group A	Low symptoms	mMRC 0-1 or CAT <10	0-1 moderate exacerbations; no hospitalization	0 (0)
Group B	High symptoms	mMRC ≥2 or CAT ≥10	0-1 moderate exacerbations; no hospitalization	30 (37.5)
Group E	Any symptom level	mMRC or CAT (any score)	≥2 moderate exacerbations or ≥1 exacerbation requiring hospitalization	50 (62.5)

The patients underwent routine biochemistry investigations and evaluation for inflammatory markers (Table 3).

**Table 3:** Baseline biochemical parameters of the study participants Values are written in mean ± SD. AST (SGOT): aspartate aminotransferase (serum glutamic oxaloacetic transaminase); ALT (SGPT): alanine aminotransferase (serum glutamic pyruvic transaminase); ALP: alkaline phosphatase

Investigation	Unit	Normal Reference Range	Mean ± SD
Procalcitonin (PCT)	ng/mL	<0.1 ng/mL	1.71 ± 1.34
C-reactive protein (CRP)	mg/L	<50 mg/L	56.78 ± 36.10
Total leukocyte count (WBC)	×10^9^/L	4.0-11.0 ×10^9^/L	11.86 ± 2.23
Hemoglobin	g/dL	12-16 (female), 13-17 (male)	14.2 ± 2.1
Platelet count	×10^9^/L	150-450 ×10^9^/L	278 ± 65
Random blood sugar	mg/dL	110-200 mg/dL	102 ± 12
Fasting blood sugar	mg/dL	70-110 mg/dL	99 ± 6
Blood urea	mg/dL	15-40 mg/dL	23.3 ± 6.6
Serum creatinine	mg/dL	0.6-1.2 mg/dL	0.8 ± 0.1
Serum bilirubin (total)	mg/dL	0.2-1.2 mg/dL	0.6 ± 0.1
AST (SGOT)	IU/L	<40 IU/L	32 ± 3
ALT (SGPT)	IU/L	<40 IU/L	31 ± 2
ALP	IU/L	40-130 IU/L	78 ± 5

When we determined the association of inflammatory markers with GOLD disease grading system, we found that compared to category B, category E had significantly higher PCT (2.69 ± 0.51 vs. 0.07 ± 0.01 ng/mL, p < 0.0001), significantly higher CRP (83.52 ± 11.84 vs. 12.2 ± 4.95 mg/L, p < 0.0001), and significantly higher WBC (13.1 ± 1.9 vs. 9.9 ± 1.1 ×10^9^/µL, p < 0.0001) (Table 4).

**Table 4:** Association of PCT, CRP, WBC with GOLD grading system Values are written in mean ± SD. * p < 0.05 was considered statistically significant. An independent t-test was applied for the comparisons (t-value). PCT: procalcitonin; CRP: C-reactive protein; WBC: white blood cells; GOLD: Global Initiative for Chronic Obstructive Lung Disease

Parameter	B (n=30)	E (n=50)	Test Value (t-value)	P-value
PCT (ng/mL)	0.07 ± 0.01	2.69 ± 0.51	36.4	<0.0001*
CRP (mg/L)	12.2 ± 4.95	83.52 ± 11.84	37.1	<0.0001*
WBC (×10^9^/µL)	9.9 ± 1.1	13.1 ± 1.9	10.0	<0.0001*

## Discussion

In our study, patients in GOLD category E demonstrated markedly higher inflammatory markers than those in category B, including significantly elevated PCT, CRP, and WBC levels. This pattern reflects a stronger systemic inflammatory response in patients with acute exacerbations, as seen in category E patients, as against category B patients who were without any acute episode of exacerbation at the time of evaluation of markers.

Similar trends have been documented in previous research. Bhardwaj et al. observed that levels of CRP and PCT were markedly higher during AECOPD and showed a gradual reduction as patients improved during discharge and follow-up, highlighting a clear relationship between disease severity and the extent of systemic inflammation [14]. Abd El Halim and Sayed also observed that serum PCT levels are elevated in AECOPD patients (1.44 ± 0.542 ng/mL) than stable COPD patients (0.05 ± 0.012 ng/mL) and healthy controls (0.04 ± 0.010 ng/mL) (p < 0.001) [15]. Even Borsi et al. found that the mean PCT level in the exacerbation group was significantly higher at 0.272 ± 0.586 ng/mL compared to 0.066 ± 0.027 ng/mL in the stable group (p = 0.001) [16].

The findings show that acute exacerbations are often accompanied by increased inflammatory markers, reflecting disease progression and greater physiological stress [17]. TLC also tends to rise with increased severity as per the GOLD disease grading system, further emphasizing immune activation in severe COPD [14]. Overall, inflammatory markers showed a significant association with acute exacerbations, differentiating categories B and E of the GOLD disease grading system.

Furthermore, it must be discussed here that from a cost perspective, CRP and TLC are inexpensive, widely available, and routinely performed investigations, making them highly cost-effective in resource-limited settings. Although PCT is relatively more expensive, its use can reduce unnecessary antibiotic exposure and hospital stay, thereby offering indirect cost benefits [18]. Moreover, these biomarkers are not intended to replace spirometry. Spirometry remains the gold standard for diagnosing and staging COPD, but is not feasible or reliable during acute exacerbations due to patient distress and poor effort [19]. In contrast, inflammatory biomarkers provide real-time information during AECOPD, making them complementary rather than superior to spirometry in the acute setting [7-11].

Regarding prognosis, elevated levels of CRP, PCT, and TLC have been associated with increased severity of exacerbation, longer hospital stay, need for ventilatory support, and higher short-term mortality, indicating their prognostic relevance in AECOPD [20,21]. Once an exacerbation is identified, management of patients with AECOPD focuses on early stabilization, control of hypoxaemia, and reduction of airway inflammation. Patients are treated with high-dose inhaled bronchodilators (short-acting β2-agonists with or without anticholinergics), systemic corticosteroids (oral prednisolone 30-40 mg) to reduce treatment failure, and antibiotics when purulent sputum suggests bacterial infection. Controlled oxygen therapy targeting SpO_2_ of 88-92% is emphasized to avoid hypercapnia. Non-invasive ventilation (NIV) is initiated early in patients with respiratory acidosis or worsening distress, significantly reducing the need for intubation and mortality [22].

Overall, the role of these markers in the routine evaluation of acute exacerbations in COPD remains justified, primarily, to identify the high-risk patients and to better manage them to improve prognosis [21]. Even the subclinical parenchymal lung injury that is present during acute exacerbations, remaining undetected on chest X-ray imaging, can be reflected by the associated inflammatory response or elevated biomarker levels [21].

Limitations of the study

This study has certain limitations that merit consideration. First, the sample size was relatively small, as the study primarily focused on patients classified under GOLD 2023 category E, which may limit the statistical power and generalizability of the findings. Second, although inflammatory biomarkers were evaluated, a formal cost analysis of these investigations, particularly PCT testing, was not performed, and therefore, cost-effectiveness could not be assessed.

Third, the study design was cross-sectional, and no longitudinal follow-up was undertaken; hence, changes in biomarker levels over time, their relationship with clinical outcomes, and their role in predicting future exacerbations could not be evaluated. Additionally, treatment modalities, therapeutic response, and clinical outcomes such as duration of hospital stay, need for ventilatory support, or mortality were not assessed.

Furthermore, potential confounding factors, including age, smoking exposure, baseline medications, duration of COPD, infection status, and coexisting comorbidities, were not adjusted for in the analysis. Smoking status was recorded qualitatively as current, former, or non-smoker, and quantitative assessment using pack-years or smoking index was not uniformly available, limiting evaluation of dose-response relationships.

Despite these limitations, the study provides preliminary insights into the association of inflammatory biomarkers with AECOPD, highlighting the need for larger, prospective, and multicentric studies with comprehensive adjustment for confounders and outcome assessment.

## Conclusions

In conclusion, AECOPD is associated with heightened airway and systemic inflammation, reflected by changes in inflammatory biomarkers such as WBC count, PCT, and CRP. Higher levels were noted in patients with acute exacerbation (GOLD category E) compared to those with less severe disease and without any acute exacerbation at the time of enrolment in the study (category B), indicating their potential role as biomarkers. These findings highlight the relevance of PCT, CRP, and WBC count in reflecting inflammatory burden and support their use as adjunctive tools in the clinical assessment and management of patients with COPD.
